# Reproductive choices: a qualitative study of Dutch Moroccan and Turkish consanguineously married women’s perspectives on preconception carrier screening

**DOI:** 10.1186/s12905-018-0574-4

**Published:** 2018-05-31

**Authors:** Petra Verdonk, Suzanne Metselaar, Oka Storms, Edien Bartels

**Affiliations:** 1 Department of Medical Humanities, Amsterdam Public Health research institute, School of Medical Sciences, Boelelaan 1089a, 1081 HV Amsterdam, The Netherlands; 2 Department of Medical Humanities, Amsterdam Public Health research institute, Boelelaan 1089a, 1081 HV Amsterdam, The Netherlands; 3 Department of Social and Cultural Anthropology, VU University, De Boelelaan 1105, 1081 HV Amsterdam, The Netherlands; 4 MOVISIE Netherlands Centre for Social Development, Catharijnesingel 47, 3511 GC Utrecht, The Netherlands

**Keywords:** Consanguinity, Preconception carrier screening, Reproductive choices, Premarital screening

## Abstract

**Background:**

Cousin marriages, in the Netherlands most frequently between Turkish or Moroccan couples, are at higher risk of having offspring with recessive disorders. Often, these couples not perceive or accept this risk, and it is hardly considered a reason to refrain from family marriages. Preconception carrier screening (PCS) is offered to Jewish groups, and more recently in the Netherlands, to genetically isolated communities. In this study, Dutch Moroccan and Turkish women’s perspectives on preconception carrier screening (PCS) and reproductive choices were explored.

**Methods:**

Individual interviews were held with Dutch Turkish and Moroccan consanguineously married women (*n* = 10) and seven group discussions with Turkish and Moroccan women (*n* = 86). Transcripts and notes were analyzed thematically.

**Results:**

All women welcomed PCS particularly for premarital genetic screening; regardless of possible reproductive choices, they prefer information about their future child’s health. Their perspectives on reproductive choices on the basis of screening results are diverse: refraining from having children is not an option, in vitro fertilization (IVF) combined with pre-implantation genetic diagnosis (PGD) was welcomed, while prenatal genetic diagnosis (PND), termination of pregnancy (TOP), in vitro fertilization with a donor egg cell, artificial insemination with donor sperm (AID), and adoption, were generally found to be unacceptable. Besides, not taking any special measures and preparing for the possibility of having a disabled child are also becoming optional now rather than being the default option.

**Conclusions:**

The women’s preference for PCS for premarital screening as well as their outspokenness about not marrying or even divorcing when both partners appear to be carriers is striking. Raising awareness (of consanguinity, PCS and the choice for reproductive options), and providing information, screening and counseling sensitive to this target group and their preferences are essential in the provision of effective health care.

## Background

In the Netherlands, preconception screening (PCS) is offered and studies are conducted to assess preferences for targeted carrier screening among Jewish groups and, more recently, among genetically isolated communities [1, 2]. When partners carry a same genetic mutation for autosomal recessive disorders they have a 1 in 4 chance of having an affected child. In clinical genetics, a consanguineous marriage is defined as an intra-familial union between people who are second cousins (fifth-degree relatives) or closer related family members [3, 4]. These couples more often have identical DNA inherited from a common ancestor. Hence, they are a target group for preconception carrier screening (PCS) also for rare diseases beyond already identified and highly prevalent recessive disorders such as thalassemia [3, 4]. In the Netherlands, where this most frequently concerns couples of *Moroccan* and *Turkish* descent, consanguinity is a sufficient indication for genetic counseling [3]. Disseminating genome-based information to an ethnically diverse audience demands reflection on intercultural communication and ethical questions. Besides, genetic technology can affect women differently than men as women feel, and are held, more responsible for the well-being and health of children than their partners [5]. Not much is known about the perspectives of consanguineously married Turkish or Moroccan women on screening, counseling and the reproductive options and choices available to them. Currently, improved risk assessment tools are being developed to identify larger numbers of pathogenic mutations (including rare mutations) and differentiate between high-risk and low-risk couples [3]. For this study, we have considered the most common reproductive options that follow upon an unfavorable outcome of PCS: (1) refraining from having children; (2) termination of pregnancy (TOP) after prenatal genetic diagnosis (PND); (3) in vitro fertilization (IVF) combined with Pre-Implantation Genetic Diagnosis (PGD); (4) in vitro fertilization with a donor egg cell; (5) artificial insemination with donor sperm (AID); (6) adoption, or (7) not taking any special measures and/or preparing for the possibility of having a disabled child.

Turkey and Morocco are both populated by Islamic majorities [6]. An important perceived advantage of consanguineous marriages is the elimination of social risk. It offers security for women and children by strengthening of family ties [7–9]. Although the discussion on medical risk for offspring in the case of marrying within the family is widespread, genetic literacy is low [8, 10, 11]. Besides, these groups hardly perceive or accept a medical risk, or do not consider that risk a reason to refrain from family marriages. Medical risk is not prioritized, as it is often considered just one of many risks in life or it is framed in terms of fate [8]. When both risks are calculated, the medical and the social, the choice to eliminate social risk gets priority.

In the Netherlands, 80% of Turkish and Moroccan migrants (who make up about 11% of the Dutch population) marry a within-ethnic group partner [12, 13]. The prevalence of consanguineous marriages among these groups is about 20–25% [14]. In the general population, a couple’s statistic risk of having offspring with severe autosomal recessive (AR) genetic disorders is 2–3%, whereas on average consanguineous couples have an additional 2–3% risk, thus 4–6%. However, only a minority (10–12%) of consanguineous couples have an increased risk of 25% or higher and thus the other 80% has a similar risk as non-consanguineous couples [3, 4]. This is related to the possibility that both parents are carriers which is higher for consanguineous couples. The most common autosomal recessive (AR) hereditary disorders among Dutch ethnic minorities are haemoglobinopathies (HbP), such as thalassemia and sickle cell disease [15].

In many Middle Eastern and Mediterranean countries, PCS is offered premaritally on a large scale, especially where both the prevalence of these AR disorders and consanguinity are relatively high [9, 16]. Although new, mostly rare, AR diseases are identified continuously, screening programmes focus on a number of diseases which are highly prevalent among the population. They offer people the possibility of considering the risks of consanguinity for offspring and, to a lesser degree, making informed choices, such as taking preventive measures [17]. Some forms of screening are mandatory, for instance the test for HIV, hepatitis and thalassaemia before civil marriage in Turkey. However, rather than providing the results of these tests, providing proof of being tested is obliged. Besides, religious marriages without having a civil marriage are still practiced frequently. In Morocco genetic tests are hardly available. Nevertheless, in an anthropological field study in Morocco and Turkey [6], all people were very well aware of the discussion on family marriages and the genetic risk for offspring but most did not perceive and did not accept the medical risk as priority.

Earlier research shows that in the Netherlands, Dutch Turks and Moroccans find PCS acceptable for severe disorders because it is perceived to provide information about the health of future children [10, 11]. Van Elderen et al. explored the acceptability of PCS in general for Dutch Moroccans, Turks and Surinamese and they did not find an association with education, religion, or consanguinity [18]. Thus, in general, primary prevention of diseases in the form of PCS seems welcomed. Less is known, however, about how this prevention is perceived by Dutch Moroccan and - Turkish women and more specifically, *consanguineously married* women, and how they welcome reproductive choices. To our knowledge, there are no studies that focused on consanguineously married women’s preferences with regard to PCS and several reproductive choices, even though more technologies are going to be available to estimate risk. Little is known about how to deliver health care to particular migrant groups and how to accommodate to their needs, but good preconception care requires insight into target groups’ perspectives, preferences and choices. Beyond diseases with a high prevalence, PCS technologies are also expected to identify carrier couples for rare AR diseases. With this study we aimed to gain a better understanding of the perspectives of Dutch Moroccan and Turkish women and, more specifically, consanguineously married women among them, on what PCS is expected to offer even when they do not perceive or accept a medical discourse on risk. Therefore, exploring the possible future implications of PCS in terms of reproductive and related choices is relevant, especially for these women.

Therefore, a key concept in our study is *frameworks of choice.* It indicates how people perceive choice, how choices are embedded in broader contexts, and how they are influenced by individuals, families and communities and are related to socio-economic circumstances, opportunities, belief systems and social networks [19]. Framing refers to the selection of aspects of reality which are important in the interpretation, communication, and reception of (social) knowledge. A process of framing takes place to bridge the ‘know-do’ gap, the gap between what is known and what is done [20]. Frameworks of choice is not opposed to the concept of *individual choice* but it “puts into perspective approaches that regard ‘individual’ or ‘autonomous choice’ [main concepts in classical bioethics] as the basis of predictive and reproductive decision-making” [19]. Understanding the frameworks of choice of individuals makes it possible to contextualize their choices. Such an approach takes into account the women’s individual agency, their social interdependency or ‘relational autonomy’ [21], and the structural gendered cultural and religious contexts in which the women’s choices are embedded. In this study we explore Dutch Moroccan and Turkish women’s perspectives on partner choice, marriage, having children, PCS and reproductive choices as embedded.

## Methods

Despite differences such as societal and ethnic background and differing interpretations of Islam, Dutch Turkish and Moroccan women have similar characteristics such as a simultaneous arrival in the Netherlands, class background, and patterns of partner choice [22]. Both groups have a relatively high rate of consanguineous marriage and in both groups, women are considered responsible for procreation and care for children. Besides, women compared with men in general have more contact with their doctors because of reproductive care. Hence, we drew from both groups and interviewed women only with the exception of one interview with a young couple. Ten in-depth interviews were conducted by OS in Dutch to explore consanguineously married women’s perspective(s) towards (a) the possibility of screening, (b) the reproductive options available afterwards, and (c) the contexts and embeddedness of their choices. We used purposive sampling to maximize the richness, depth and variability of the data and selected a variety of individual interviewees, based on a marriage with a cousin, having children or not, disabled children or healthy, age (ranging from 24 to 50 years old), and Turkish and Moroccan background (Table 1, pseudonyms used) [23]. Since the interviews were held in Dutch, all women had to be able to speak Dutch sufficiently. For this article, quotes from the interviews were translated in English. Women were recruited through Turkish and Moroccan women’s organizations, a self-support group for mothers with a disabled child via a care organization, and through students. During one interview the husband was present (Meryem and Kerem). In such case, concern is warranted whether and how the presence of their partner influences the participants’ freedom of expression. However in this interview, both partners were very open to discussing moral dilemmas and the reproductive choices they made, as shown in our results section. Interviews were held in the women’s homes and took approximately 2 h.

**Table 1:** Overview of Individual Interviewees’ Characteristics

No.	Name^a^	Education	Ethnic back-ground	Migrant status (1st or 2nd generation, age of migration)	Children	Disability/disease^b^
1	Nawel	Highly educated	Moroccan	1st generation, age 29	yes	Two children diseased Genetic risk unknown
2	Kaoutar	Intermediate	Moroccan	1st generation, age 21	yes	Child diseased Genetic risk present
3	Nesrin	Highly educated	Turkish	2nd generation	yes	Child diseased Genetic risk unknown
4	Meryem (& Kerem)	Intermediate	Turkish	2nd generation	yes	Two children diseased Genetic risk present
5	Aysel	Intermediate	Turkish	2nd generation	no	Child desired
6	Gülşen	Intermediate	Turkish	2nd generation	yes	
7	Farida	Low	Moroccan	1st generation	yes	One child diseased Genetic risk present
8	Malika	Low	Moroccan	1st generation	yes	
9	Amade	Intermediate	Turkish	1st generation	yes	
10	Sarah	Intermediate	Moroccan	2nd generation	yes	

^a^Pseudonyms

^b^Disability/disease not specified; the information is based on mothers’ evaluation of the disease and its genetic risk

We were interested in the possible choices and the contexts in which meaning making on PCS and reproductive choices is produced. Starting point is the notion that health care decisions are made and embedded in daily life. Discussions in ‘natural’ groups of people, people who know each other already, is useful to gain insight in the content of social knowledge about topics such as consanguineous marriage, PCS, and reproductive choices, and to access how that knowledge is generated, framed, and embedded within possible choices. These discussions help to gain insight in an agreed upon perspective on consanguineous marriage, PCS, and reproductive options shared group culture [23]. So prior, and running parallel (April 2011–January 2012) to the individual interviews (December 2011–September 2012), 7 natural group discussions with in total 86 participants, were organized by women’s organizations (2 Turkish, 3 Moroccan, and 2 mixed groups) and led by EB, an anthropologist with longstanding experience with women with these background both as migrants in the Netherlands as well as in countries of origin, and who had collaborated with the women’s organizations before, and by OS (an anthropologist and a PhD-student). The participants were informed about the research, but there was no personal relationship between researchers and participants. The women’s organizations had regular group meetings with women from the local communities. The women knew each other well and as they met regularly, they formed ‘natural groups’. Social workers and group leaders of these organizations, community members themselves knowing the population very well, approached participants personally for the individual interviews or approached women during these regular meetings. When the women gave permission for the interview, the researcher contacted them. Group meetings with the particular aim to discuss consanguinity and reproductive choice were also took place in the community centers that were run by the women’s organizations. Not all participants of these group discussions were consanguineously married and 2 participants had other ethnic backgrounds. As the women’s organizations are experienced with volunteering translators during their meetings, we trusted in these translators during the group meetings for this project. Hence, a female volunteer who could translate between Dutch-Arab, Dutch-Tamazight, as well as a female volunteer who could translate between Dutch-Turkish were present in the group discussions. The women do not have official interpreter credentials. In the group discussions and interviews the same topic list was used which was based on the literature. Participant observation was partly possible for instance during dinners with the respondents and at meetings in mosques. This offered the opportunity to learn about the background of the women and seek their views in the discussions between them. Validity was reached by triangulation of research methods, discussions among the researchers (researcher triangulation), in-depth interviews, and minutes of the group discussions (data triangulation). Fieldwork was carried out by the two anthropologists in the research team. All researchers are female.

The individual interviews were tape-recorded with the participants’ consent including the use of direct quotes in the manuscript. During the group discussions, notes were taken by the two researchers who were present. They asked for permission to record the group discussions, but not all women consented. New topics raised during group discussions were checked in interviews and vice versa and no more interviews were conducted when no new information emerged (data saturation) (see Table 2).

**Table 2:** List of Topics

Personal information	Age, educational background, migrant status, age of migration, children.
Consanguinity	Relationship to partner prior to marriage, marriage history, perspectives on consanguinity (disadvantages, advantages), consanguineous marriages in family and social environment, differences in perspectives among generations.
Genetic risk	Knowledge, perspectives on genetic risk.
Children’s health	Disease, diagnostic history, genetic background, living with a child with a disability/disease, mother’s and father’s ways of coping, experiences in health care.
Genetic testing	Perspectives on testing.
Reproductive options	The possibilities of PCS in the future: (1) not having children; (2) termination of pregnancy (TOP) after prenatal genetic diagnosis (PND); (3) in vitro fertilization (IVF) combined with Pre-Implantation Genetic Diagnosis (PGD); (4) in vitro fertilization with a donor egg cell; (5) artificial insemination with donor sperm (AID); (6) adoption, or; (7) not taking any special measures or preparing for possibly of having a disabled child.

PV hand-coded the interviews line by line. Researcher triangulation was applied to base coding and analytic decisions on convergent validation: discussions on coding, clustering, and analyzing were held among all four researchers. All the interviews were transcribed verbatim and read several times to get a feeling for the depth of the data, and to collate and discuss ideas that came up during its reading. Based on a thematic analysis approach, we identified key themes relating to PCS and reproductive options and clustered thematically to identify and report patterns and categories in the data [23]. For instance, we identified 19 *characteristics of a good husband* (code), such as trustworthiness, education, and parents’ approval. Focusing on the research question, *frameworks of choice* helped to contextualize preferences for and ambivalences towards PCS and reproductive options. Quotes presented are mainly derived from the individual interviews as they give an insight not only in what was said, but also in ‘who the women were’ and how they expressed themselves.

## Results

We identified four major themes in the women’s perspectives which we will subsequently describe: (1) partner choice; (2) marriage and having children; (3) healthy children, and; (4) having your own child.

### Partner choice

Perspectives on PCS and the options which ensue are framed and embedded within the contexts of family, religion, and gendered norms. These aspects were seen as mutually constituting and influencing each other. For a part of the women consent with the rules set within these contexts is expressed in how consanguineous marriage is ‘normal’ or even a preferable choice and how they ‘just’ stopped working after marriage. Other (especially younger) women express ambivalence towards consanguineous marriages and gender roles. Aysel (26 years), grew up in the Netherlands. Originally she opposed to cousin marriage, but then developed feelings for her cousin although she was not in love. But he just seemed ‘perfect’, and she accepted his marriage proposal. Soon she considered breaking up the relationship, continued on however, and a difficult time awaited them. After a holiday and spending time together they started getting along again. Her Turkey-raised husband got an education in the Netherlands, started his own business, and let go of his rules for her:

“And since, about four years, everything is perfectly happy. Because otherwise I would get a divorce. When it doesn’t work, it was just a black life that I see before me. Just, one and a half year, a black life. I could not live like that my entire life. And he says the same thing. He gave me time. He says Aysel, look, when you say ‘this does not work’ then it does not work.” (Aysel).

The ideal for all of them seems that a couple should be ‘able to live a life together’ or compatible, and husbands should meet criteria such as an education, work, parents’ consent, or not using drugs. Most important and always mentioned was being a Muslim as a precondition for a successful marriage: *“If all men follow the Prophet’s example, everything goes well”* (Group discussion). In case of an arranged marriage, most women hoped to fall in love with their partners but other women fell in love with their cousins first before entering marriage. Partner choice based primarily on love seems more important for the second generation migrant women than for the first generation. For all women, in arranged or love marriages, cousins are only eligible if they are men with whom the women had little contact before marriage, often because they live far away in the country of origin. ‘Real cousins’, i.e. kin that you actually grew up with and who ‘feel like a brother’, are not eligible to marry [8], and the Moroccan group leader of one of the group discussions expresses this widely held belief as follows: “How to marry someone with whom you played in the sandpit?”

Forced marriage was rejected by all women. But in practice some women had either experienced or witnessed both covert and overt coercion. In any case, family and parents are heavily involved in partner choice, either by arranging or consenting to a marriage.

Most women felt they could have refused a proposed spouse and, therefore, believe they have individual autonomy in partner choice. The women perceive that men are granted more freedom than women, but the younger generation seems to claim and gain more and more ground for individual partner choice. Nevertheless, approval of parents remains highly important for most women [8].

### Marriage and having children

Malika finds PCS unnecessary ‘because everything is God’s will’, but all the other women were positive about PCS, mainly to have *certainty* about the child’s health. Kaoutar, (divorced) who has a child with a severe disability, favours PCS for a possible next child with a possible next husband:

‘To have a clear picture. With him [ex-husband, cousin], I do not know what caused it. Yes, I’d just want to know.’ (Kaoutar).

Furthermore, although the women desire healthy children and intend to participate in PCS, they consider the genetic risk related to consanguinity to be low. Consanguineous couples can have healthy children and non-consanguineous couples can have disabled children, they argue; other factors cause health problems too. Genetic risk does not seem to play a role in consanguineous partner choice, and may even be denied. This does not conflict with their interest in PCS. PCS offers the possibility to get information on the health of the child, about compatibility with the partner, or about reproductive potential (as also found by e.g. [9]). Children are considered to be central to a marriage; refraining from having them is out of the question because *‘you want to feel like you are a family’* (Gülşen). Aysel literally does not want to think about the question: *‘All those strange questions that I cannot answer! I really do want a child.’* Farida, who chairs a women’s organization and hears of illegal polygamy cases in the Netherlands, explains how actively refraining from having children for carrier couples can be risky for women:

‘You have to know for sure that this man can be trusted, that he remains faithful. Generally, when you are in love, everything is okay, but after that period, there might be a second wife, as he has a right to take a second wife if he wants children.’ (Farida).

Therefore, not marrying a carrier partner is a viable option and for some divorce is preferable to refraining from having children. Aysel, hoping to get pregnant for several years now, would not have married her cousin if they were a carrier couple. For women in love marriages, breaking up the relationship is not an option. Gülşen (25 years, arranged marriage) balances avoiding and accepting the risk of having a child with a disease while embracing PCS itself:

‘I do not think that someone, when she hears that she has 25% chance to have a baby with a genetic disease, will say no, when she wants to marry. When they really love each other. But when that woman says no, the risk is too large so I will not marry, that is possible too. It can work in both ways. It is a choice and about risk assessment.’ (Gülşen).

Being in love with your partner is considered a *dis*advantage for a carrier couple, but an arranged couple *should* stop seeing each other, which is what Gülşen would do because:

‘When a child is ill during the night, as a mother you are completely stressed out. Measuring their temperature, when they have diarrhoea you have to take care of them. And when a child will have a disease it is really not worth it to marry someone, I would not do it. (…) They think let’s do it because it is a small risk. But later when they realize it when they have it [the child] they regret it, because it is not worth it. It will destroy your marriage.’

However for Gülşen, a genetic risk up to 25% is not high enough to worry about, because it does not provide enough certainty about the child’s health. Rather than having PCS after marriage, the women do prefer premarital screening and carrier status to be added to the long list of criteria for eligible husbands.

### Healthy children

If a carrier couple wants to have (more) children together, termination after PND or IVF with PGD are possible options. Both technologies put a heavy burden on women, but with PND/termination, the women perceive loss, such as transgressing religious rules, the difficulty of letting go of a pregnancy, and uncertainty about the future. With IVF/PGD, they perceive only gain, i.e. *certainty* about the child’s health. For some women, termination is taboo and forbidden (‘haram’), for others, it is acceptable if the mother’s life is endangered or the child’s diagnosis is severe. Religious permission is decisive but the women seem uncertain about what is allowed:

‘Abortion is the last option I would consider. I try to live by my faith. […] I heard that before the child is one and a half month you can have an abortion when something is wrong. After that period, not anymore. But I have only heard this, I do not know for sure now. First, I would check whether that is allowed by my faith. If it is allowed, I would do it.’ (Aysel).

Meryem and Kerem are a carrier couple, and after their first baby died from an AR disease they opted for PND and possibly, termination, in the second pregnancy. Tragically, Meryem’s second baby also died but from yet another AR disorder which had not been tested during pregnancy. They went to Turkey for IVF after PGD. Rather than a burden, it felt a bit like a holiday to her, in-between the treatments. Now, she has healthy twins and states that IVF/PGD provides ‘certainty’:

‘Suppose you get pregnant, and you want to terminate it, that is really hard. So you’d rather have a selection at the start, and then have it placed back. Then you know that it all goes well. It is just 98, 99% certain that everything goes well.’ (Meryem and Kerem).

In contrast to abortion, the women are certain that Islam allows IVF/PGD, although Islam offers no precise definition of the beginning of life, the moment of conception, and the onset of ensoulment. When couples do not wish to separate, IVF/PGD as a preventive measure seems acceptable because an actual pregnancy is not yet established as Gülşen explains:

‘No, then it is not yet a child, only when the brain is developed and so on. Actually, from the moment of conception it is a child. But the organs are not developed yet.’

When a woman is pregnant, the child is accepted. Having an affected child is an assignment from God, not a punishment, but rather the contrary, according to Kaoutar: an affected child is for selected people exclusively, for those with *sabr,* patience, energy, and power. In the end, most women agree: ‘How we are made is God’s will’.

### Having your ‘own’ child: Biological and social parenthood

For most women, a child should be genetically one’s own and born within marriage. A donor gamete is ‘not your own child’. Despite the flexibility she perceives in her faith, Farida stressed that:

‘I do not think that anyone will do that. It is not your real child. And it is not allowed by our faith. Whether it is a man’s or a woman’s gamete, it does not matter. You must be married to have children.’ (Farida).

Nesrin, however, assumes that couples would differentiate between egg cells and sperm. Her remark exemplifies how the women perceive double standards for men and women, not as large inequalities, but small and sensible, ‘just the way it is’:

‘I think that for mothers, they will be less difficult, because the fathers’ sperm is more important. Because he is the namegiver. So yeah, it doesn’t matter which mother. If only the name of the father is continued. [I: And what do you think yourself?] No, I do not think so. [I: For the same reasons?] No, I would not…no. I would not like it. Even when I know it is for medical reasons, it almost sounds like adultery.’ (Nesrin).

Like donor gametes, adopted children are ‘not your own’. Although adoption is preferred over donor gametes, complications may arise as the adopted child grows up:

‘I would really love to. And so does my husband. But, when I adopt a girl, after a while she is no longer […] halal for my husband. And then we live in the same house and she must cover herself all the time and she must pay attention to everything and she is just not ours.’ (Aysel).

Both the use of a donor gamete and adoption do not result in having ‘your own child’, but whereas ambivalence exists towards adoption, the use of donor gametes is clearly refuted.

## Discussion

A complex mix of religious, secular, cultural and gender logics frames the women’s perspectives on PCS and their reproductive choices. In line with earlier reports, the Dutch Turkish and Moroccan women in our study welcome PCS [10, 11]. Above all, they prefer information about their future child’s health. A Dutch anthropological study concluded that, according to Dutch Muslim theologians, imams and physicians, couples would not be interested in PCS because they either consider the genetic risk to be low or have religious reasons and choices to refrain from screening [24]. But in close agreement with Dutch Muslim theological expert opinions that in Islam, pursuing health and gaining knowledge through science are important, our study reveals that the women *do* want to know.

Second, the women’s outspokenness about not marrying or even divorcing when both partners are carriers is striking, as is their preference for PCS for premarital screening. Marriage should not endanger the health of future children [24].

Third, the difference in attitude towards PND/termination of pregnancy and IVF/PGD is remarkable. For some women PND/termination is taboo and forbidden (‘haram’). For others, it is a serious option to consider under rare conditions. In any case, religious permission is decisive. Women’s insecurity about Islamic perspectives towards termination of pregnancy has been reported earlier [25, 26]. Other studies also show that termination of pregnancy is hardly acceptable to migrant Muslim women [10, 11, 25], although reproductive choices including termination of pregnancy, may be highly dependent upon the particular diagnoses [27]. The women were very positive about IVF/PGD, because they expect it to provide certainty about a future child’s health. Interestingly, they did not mention the burden for the mother, the artificiality of conception, or the health risks. Rather, they stressed the fact that these procedures take place outside of the womb, and are therefore acceptable interventions and in line with supposed religious prescriptions.

Fourth, reproductive technologies such as PCS, IVF and PGD, are considered to be valuable particularly because they are regarded by the interviewees as interventions that provide certainty about the health of a future child. For our interviewees, refraining from or avoiding future procreation is not an option. Especially since having children is socially required; it is grounded in family values and gender norms [28].

Finally, gamete donation and adoption, are considered less acceptable because the women want to have ‘their own child’. Not doing anything or preparing for a diseased child are hardly discussed, since they seem to be the default option: when a woman is pregnant, a child is accepted as it is.

Our findings raise several concerns. Most of our interviewees had no individual experiences with PCS and its consequences. They emphasized their own agency but also explained how their decisions are and would be embedded and framed within gender roles, religious and cultural factors. Our interviewees, for instance, did mention faith, gender inequalities such as polygyny or the greater freedom that men have in choosing a partner. The concept of frameworks of choice is relevant here. On the other hand, from a health care perspective, respecting the women’s autonomy, means that one is obliged to promote it [21]. Women are confronted with different reproductive choices than men and face different consequences [5]. Our interviewees did not mention the possibility of *being refused* as marriage partners; they only considered the possibility *of refusing;* conceptions that genetic or biological ancestry is ‘stronger’ through paternal than maternal lines, may explain their views [29, 30]. But carrier status may stigmatize women in particular, labelling them as non-eligible wedding partners within their communities [15]. When screening and the disclosure of the results cause problems for women with positive carrier status, they face either the risk of having a child with a disability, or of being an unmarried outsider. The women we interviewed do not expect problems, possibly because healthy children are so important to the family. But not marrying or divorcing means that the women have to share their genetic information with their families. Pakistani adults in the UK would not readily share genetic information within the family as Shaw and Hurst [30] explain; information was kept private for reasons related to disruptive effects. In our study, the women did not refer to these consequences of disclosing genetic information. And besides possible stigmatization, people also individually need to come to terms with being a carrier [31]. New reproductive technologies may change the women’s social interdependency and their social embeddedness as is already known from other technologies. In particular contraceptive measures have been revolutionary in changing women’s and men’s lives before.

There are several limitations to our study which requires further research. First, the findings must be further verified in larger studies. We spoke with only ten individual women and seven groups; partners, families, and unmarried individuals were not included. Second, as found in other studies, in reality the women may be more positive towards termination of pregnancy [10] and more negative towards IVF/PGD [31]. Third, effectively conveying information about genetic technologies during the interview may have been problematic because genetic literacy is low [18, 19]. Besides, the costs of technologies in the interviews has not been discussed. Fourth, all the women spoke Dutch and were prepared to speak with us about this subject, although we involved a translator in our group discussions.

Our study has several implications. In general, more awareness seems useful about consanguinity, genetic risk, and counseling. Misunderstanding occurs because on the one hand consanguineous couples underestimate their genetic risks, although they do want to know what these risks are, while on the other hand, primary health care providers in the Netherlands think that consanguineous couples do not wish to discuss these issues. In addition, many primary health care providers disapprove of consanguineous marriage [3].

Second, information can be provided about PCS and IVF/PGD, and about the limitations that these technologies have. The genetic variants that are found may have unknown effects and not all possible disorders are tested for, but people may feel reassured of having a healthy baby after PCS and/or IVF/PGD.

Third, PND/termination was mostly refuted because of religious beliefs, but health care providers should still discuss it [25, 32]. Different Islamic viewpoints do exist about termination of pregnancy, which may be mentioned by family physicians or theological experts [32]. For instance, according to some Islamic scholars termination is permissible in case the child has a severe condition.

Finally, our study has shown that PCS seems welcomed in particular before marriage. Health services can help individuals to make choices in the view of their specific risks and frames of choices as related to family goals, ethical and religious values, and to act in a manner which supports and confirms their choices [4, 29, 33].

However, implementing premarital screening is challenging. Generally, couples-to-be do not present themselves as such to health care providers. Both health care providers and consanguineous couples themselves consider the genetic risk to be low. Moreover, offering premarital screening largely relies on the subjects identifying their own ethnicity and consanguinity, which does not necessarily correspond to genetic risk [34]. Finally, at this moment, the stigmatization of consanguineous couples is a realistic negative side-effect of the ethnically-targeted implementation of screening [4, 29, 35]. Currently, Dutch politicians overestimate the risk and negatively frame consanguinity, genetic risk, migration, and relate these issues to forced marriages [36]. A realistic understanding of these risks, the choices which can be made and the perceived benefits of consanguinity should therefore be encouraged among politicians and policymakers.

## Conclusion

New technologies for PCS are welcomed by consanguineously married women regardless of possible reproductive options because it provides information about the future child’s health. Their preference for PCS for premarital screening as well as their outspokenness about not marrying or even divorcing when both partners appear to be carriers is striking. Raising awareness (of both risks and options and choices that can be made), and providing information, screening and counseling sensitive to this target group and their preferences are important.

## Abbreviations

AR: Autosomal Recessive
IVF: In Vitro Fertilization
PCS: Preconception Carrier Screening
PGD: Preimplantation Genetic Diagnosis
PND: Prenatal Diagnosis
TOP: Termination of Pregnancy
